# Temporal Analysis of Hepatitis C Virus Cell Entry with Occludin Directed Blocking Antibodies

**DOI:** 10.1371/journal.ppat.1003244

**Published:** 2013-03-21

**Authors:** Marion Sourisseau, Maria L. Michta, Chati Zony, Benjamin Israelow, Sharon E. Hopcraft, Christopher M. Narbus, Ana Parra Martín, Matthew J. Evans

**Affiliations:** Department of Microbiology, Mount Sinai School of Medicine, New York, New York, United States of America; University of Washington, United States of America

## Abstract

Hepatitis C virus (HCV) is a major cause of liver disease worldwide. A better understanding of its life cycle, including the process of host cell entry, is important for the development of HCV therapies and model systems. Based on the requirement for numerous host factors, including the two tight junction proteins claudin-1 (CLDN1) and occludin (OCLN), HCV cell entry has been proposed to be a multi-step process. The lack of OCLN-specific inhibitors has prevented a comprehensive analysis of this process. To study the role of OCLN in HCV cell entry, we created OCLN mutants whose HCV cell entry activities could be inhibited by antibodies. These mutants were expressed in polarized HepG2 cells engineered to support the complete HCV life cycle by CD81 and miR-122 expression and synchronized infection assays were performed to define the kinetics of HCV cell entry. During these studies, OCLN utilization differences between HCV isolates were observed, supporting a model that HCV directly interacts with OCLN. In HepG2 cells, both HCV cell entry and tight junction formation were impaired by OCLN silencing and restored by expression of antibody regulatable OCLN mutant. Synchronized infection assays showed that glycosaminoglycans and SR-BI mediated host cell binding, while CD81, CLDN1 and OCLN all acted sequentially at a post-binding stage prior to endosomal acidification. These results fit a model where the tight junction region is the last to be encountered by the virion prior to internalization.

## Author Summary

HCV is a serious public health problem. Although new treatments have recently become available, it is clear that effective therapies will require combinations of inhibitors targeting diverse stages of the viral life cycle. While the HCV cell entry process is considered a suitable antiviral target, a lack of understanding of this process has hampered the development of inhibitors. It is widely accepted that HCV cell entry requires many cellular proteins that are used in a nonredundant and sequential manner. However, a critical piece of information supporting this model – the determination of when OCLN is used during this process – could not be addressed due to a lack of reagents that specifically target this protein. In this study, we derive mutant OCLN proteins whose HCV cell entry activity can be blocked by incubation with an antibody. These mutants allowed us to show that OCLN is used very late in the HCV cell entry process, which fits a model in which tight junction components are required later in the process than more exposed factors. Furthermore, our studies suggest that HCV virions may interact directly with OCLN, which has thus far not been demonstrated experimentally.

## Introduction

Hepatitis C virus (HCV), a member of the *Hepacivirus* genus within the family *Flaviviridae*, is the causative agent of over half of all liver cancers and responsible for the majority of liver transplants worldwide [1]–[3]. Even with the recent approval of HCV protease inhibitors, HCV directed therapies are often ineffective, associated with severe side effects, and prone to viral resistance [4], [5]. Although the HCV cell entry process is a target for antiviral development, the realization of this goal will require a greater understanding of its mechanisms.

HCV host cell entry requires the two viral envelope glycoproteins, E1 and E2, and numerous cellular factors, including the low density lipoprotein receptor (LDL-R) [6]–[9], glycosaminoglycans (GAGs) [10], [11], the high density lipoprotein receptor scavenger receptor class B type I (SR-BI, also known as CLA-1 and SCARB1) [12], the tetraspanin CD81 [13], the cholesterol absorption regulator Niemann-Pick disease type C1-like 1 (NPC1L1) protein, and two tight junction (TJ) proteins, claudin-1 (CLDN1) [14] and occludin (OCLN) [15], [16]. Experiments using reagents that conditionally block access to each cellular factor, such as antibodies and protein fragments, revealed that the HCV virion uses each in a multistep manner to eventually mediate its clathrin-dependent endocytosis and low-pH mediated fusion of viral and cellular lipid membranes in an early endosome [10], [17]–[21]. GAGs and LDL-R mediate virion binding [6]–[11], [22], SR-BI acts as either a binding [23] or post-binding entry factor [24], CD81 [10], [14], [25], [26] and CLDN1 [14], [27] play post-binding roles in the HCV cell entry process.

A major limitation of these prior HCV cell entry studies is that none have examined when OCLN acts during the HCV cell entry process. Although OCLN does not appear to play a role in virion binding [28], the lack of reagents that specifically inhibit its cell entry factor activity has prevented a more detailed examination of when this protein is required during the HCV cell entry process. In the studies presented here, we developed OCLN mutants whose HCV cell entry factor activity could be conditionally blocked and used these along with inhibitors targeting other HCV cell entry factors to examine the timing of cell culture derived HCV (HCVcc) entry in polarized HepG2 cells expressing miR-122 and CD81, which renders them able to support the entire HCV life cycle [29]. We found that OCLN acts late in the HCV cell entry process, during the post-binding stage and after both CD81 and CLDN1.

## Results

### Generation of antibody blockable OCLN mutants

Study of the role of OCLN in the HCV cell entry process required methods to conditionally regulate its ability to participate in this process. OCLN is a four transmembrane domain containing protein with intracellular termini and two extracellular loops termed EC1 and EC2. Although antibodies that bind to extracellular regions of receptors are commonly used to inhibit interaction with virions, to our knowledge all currently available commercial and research lab derived OCLN-specific antibodies recognize the intracellular domains of the protein and are thus not useful for such studies. Instead, we sought to create an OCLN mutant with an epitope sequence inserted in an extracellular region that efficiently mediates HCV cell entry, yet is inhibited in this function by an antibody targeting this epitope. This approach is similar to one we previously used to study CLDN1 entry factor mechanisms [14].

**Figure 1:**
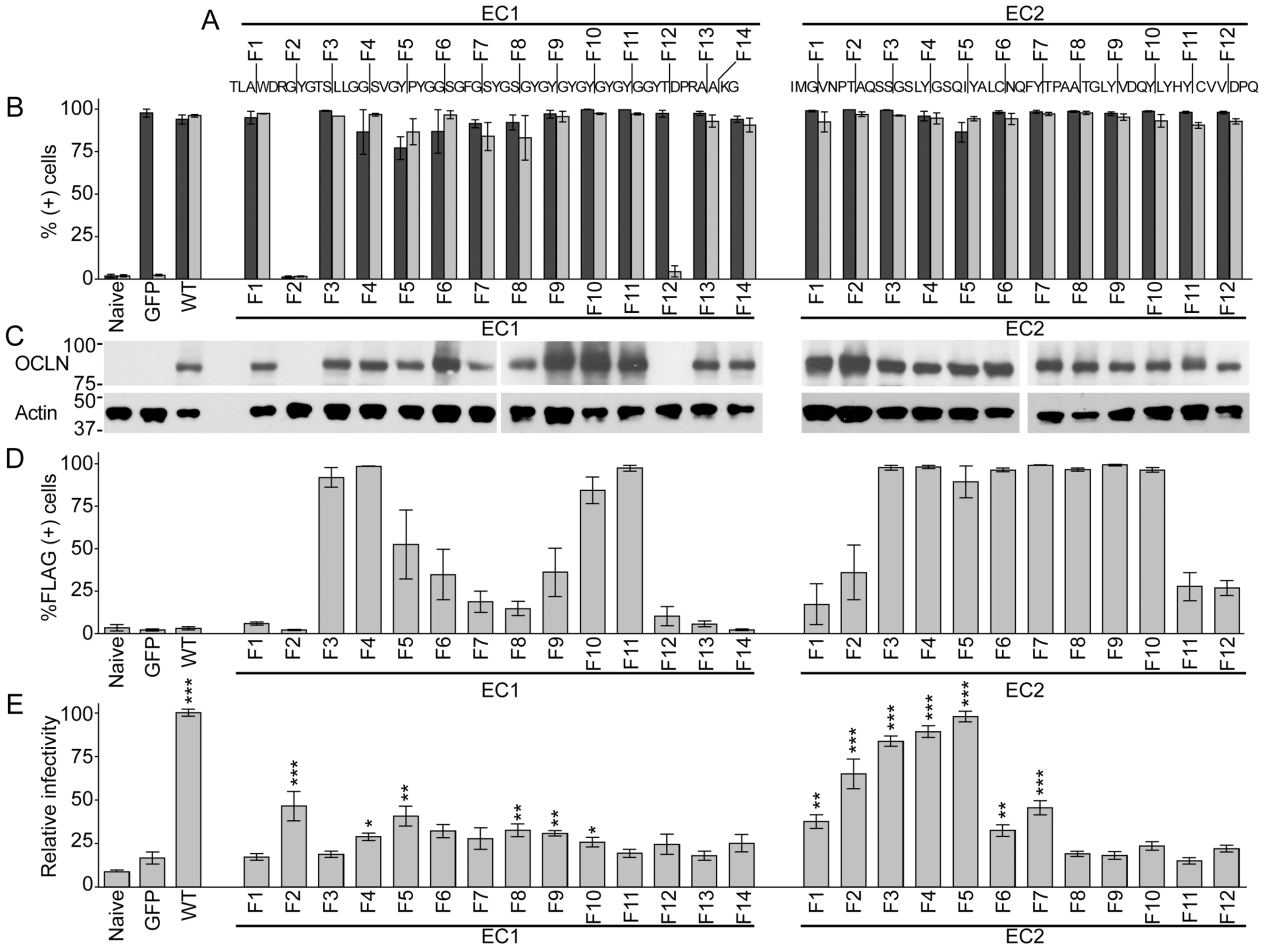
Characterization of OCLN FLAG insertional mutants. (A) Amino acid sequence of human OCLN EC1 and EC2 sequences with the location of each FLAG epitope insert marked. (B) 786-O cells were transduced with lentiviruses to express either GFP alone, wild type (WT) OCLN, or the indicated FLAG insertion OCLN mutants. These cell populations were then expanded and analyzed by FACS for expression of GFP, which is fused to each OCLN protein, (dark gray) and for OCLN expression after staining permeabilized cells with an OCLN antibody (light gray). (C) Lysates from these cells were probed by immunoblotting with either OCLN or β-actin specific antibodies. Approximate molecular weight (kDa) marker positions are indicated to the left of each blot. (D) To probe for cell surface accessibility of the FLAG epitope, transduced cells were fixed and nonpermeabilized cells were stained with the FLAG M2 monoclonal antibody. (E) These cells were then challenged with H77 genotype 1a HCVpp to determine their capacity to support HCV cell entry. ‘Relative infectivity’ refers to luciferase values measured two days post infection normalized to parallel VSVGpp infections, to control for variations in cell number, and set relative to luciferase values for infections of cells expressing wild type human OCLN. *P<0.05, **P<0.01, *** *P*<0.001 (Mann-Whitney test).

**Figure 2:**
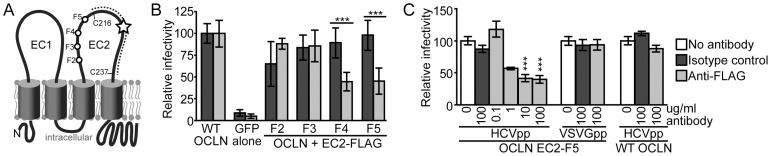
OCLN directed FLAG antibody inhibits HCVpp infection of 786-O cells. (A) Illustration of OCLN membrane topology. The region flanked by two conserved cysteine residues (C216 and C237) that was previously shown to be essential for HCV entry is marked by a dotted line and a star highlights the location of species-specific determinants of OCLN entry factor activity. F2–F5 indicate the location of the EC2 FLAG insertions that did not disrupt HCV cell entry factor activity. (B) 786-O cells expressing wild type OCLN, GFP alone, or the indicated FLAG insertion OCLN mutants were challenged with H77 genotype 1a HCVpp in the presence of 10 µg/ml of an irrelevant isotype control (dark gray) or the FLAG M2 monoclonal antibody (light gray). Results were normalized to parallel VSVGpp infections and set relative to infection of cells expressing wild type OCLN. (C) To demonstrate the specificity and dose dependence of FLAG antibody inhibition, 786-O cells expressing either the OCLN EC2-5 mutant or wild type OCLN were challenged with HCVpp in the absence of antibody (white), or in the presence of the indicated concentration of either isotype control (dark gray) or FLAG antibody (light gray). *** P<0.001 (Mann-Whitney test).

We constructed a panel of OCLN mutants with a FLAG epitope sequence inserted every four amino acids throughout EC1 and EC2, labeled EC1-F1 to F14 and EC2-F1 to F12, respectively (Figure 1A). To assay the ability of these mutants to support HCV cell entry, each was expressed by lentiviral transduction in human renal carcinoma 786-O cells, which are normally not able to support HCV cell entry due to insufficient OCLN levels [15]. In these cells, most mutants were well expressed when assessed by FACS analysis for GFP expression, which is fused to each OCLN protein, and by immunoblotting with OCLN specific antibodies (Figure 1B and C, respectively). Cell surface staining of transduced cells with anti-FLAG antibodies for FACS analysis showed considerable heterogeneity in the capacity of each mutant to display a FLAG epitope on the cell surface (Figure 1D). While cells expressing 21 of these clones exhibited more FLAG antibody cell surface staining than naïve cells, only 13 mutants resulted in greater than 50% staining of each population. However, when challenged with lentiviral particles bearing the H77 genotype 1a isolate glycoproteins (H77 1a HCVpp), only four OCLN insertion mutants (EC2-F2, F3, F4, and F5, location of epitope illustrated in Figure 2A) exhibited HCV cell entry factor activity that was within 50% of wild type OCLN (Figure 1E and Figure 2B, dark filled columns).

To determine if the HCV cell entry factor activity of these four OCLN mutants could be blocked by anti-FLAG antibodies, we next infected 786-O cells expressing either wild type or mutant OCLN with H77 1a HCVpp in the absence or presence of an anti-FLAG monoclonal antibody. While the FLAG antibody did not significantly alter the ability of HCVpp to infect wild type OCLN or EC2-F2 and F3 expressing 786-O cells, it reduced HCVpp infection of cells expressing the EC2-F4 and F5 mutants by more than 50% (Figure 2B, light filled columns). As the basal HCV cell entry factor activity was highest for EC2-F5 (Figure 1E and Figure 2B), we chose this mutant for further experimentation. We confirmed that the EC2-F5 antibody inhibition was dose dependent and HCV specific, as the maximal amount of FLAG antibody tested did not impair VSVGpp infection of OCLN EC2-F5 cells or HCVpp infection of 786-O cells expressing wild type OCLN (Figure 2C).

### OCLN directed antibody blocking is not due to OCLN internalization

**Figure 3:**
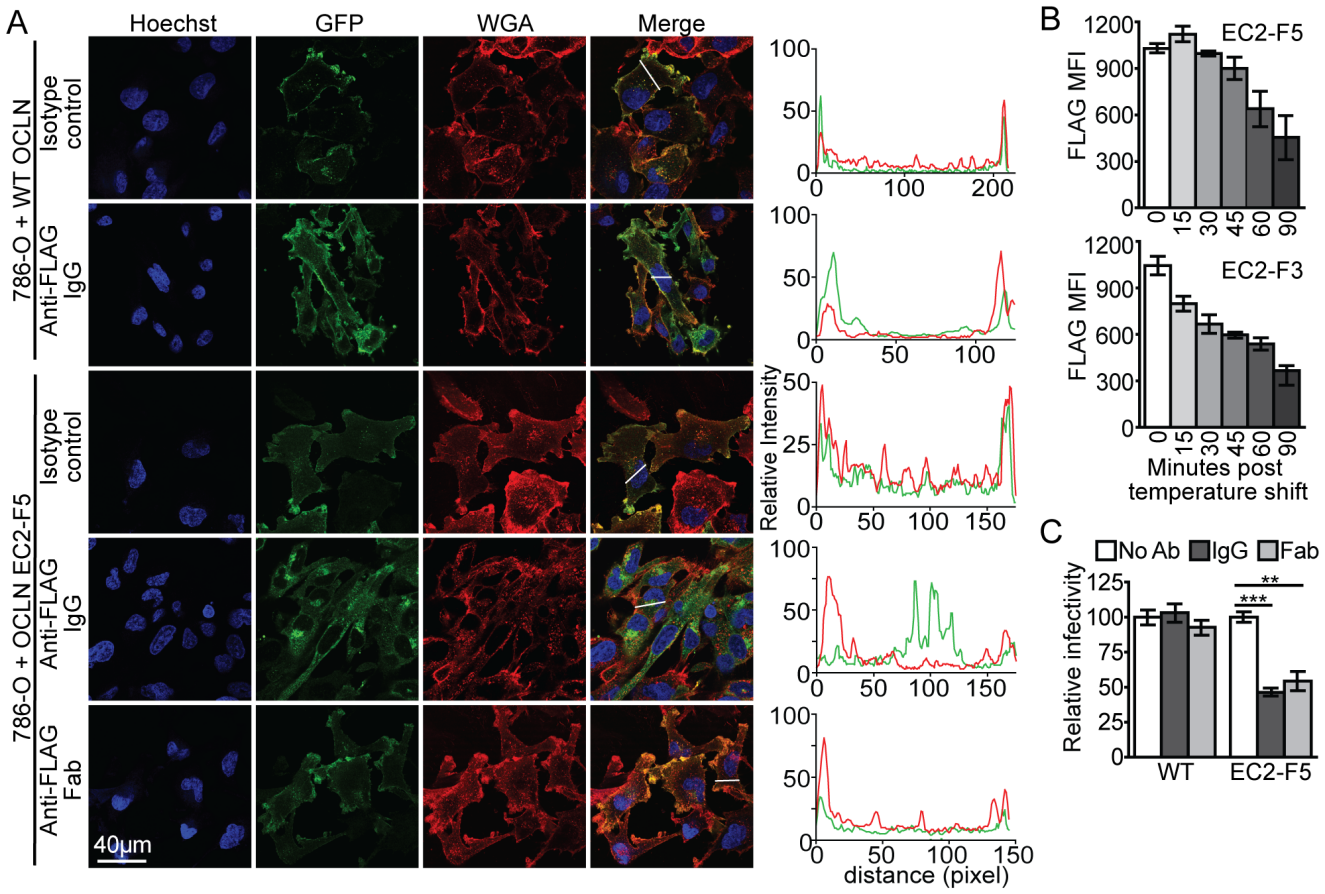
OCLN directed FLAG antibody inhibition is independent of endocytosis induction. (A) Subcellular localization, assessed by confocal microscopy, of wild type or EC2-F5 OCLN-GFP fusion proteins in 786-O cells following a two hour 37°C incubation in the presence of either 10 µg/ml isotype control, FLAG antibody, or FLAG Fab, as indicated. In each set, blue, green, and red represent Hoechst 33342 nuclear DNA staining, GFP fluorescence, and Alexa Fluor 594 wheat germ agglutinin (WGA) staining of the cell membrane, respectively. To the right of each set is a graph showing the quantification of GFP (green line) and WGA (red line) signal intensity across a representative cell cross section, indicated by the white line in the corresponding merged panel. A representative example of greater than three independent experiments is shown. (B) 786-O cells expressing either the EC2-F5 or F3 OCLN mutant were incubated with 10 µg/ml of FLAG M2 antibody for 15 min at 4°C. Antibody was then washed off, the temperature shifted to 37°C, and at the indicated time points cells were fixed and stained with secondary antibody for FACS analysis to determine surface accessible primary antibody. (C) 786-O cells expressing either wild type or the OCLN EC2-F5 mutant OCLN were challenged with HCVpp in the absence of antibody (white), or in the presence of FLAG M2 IgG (dark gray) or Fab (light gray). **P<0.01, *** P<0.001 (Mann-Whitney test).

While we hypothesized that FLAG antibody blocked the ability of the OCLN EC2-F5 mutant to participate in HCV cell entry by interfering with an interaction between OCLN and a cellular or viral protein, a second possible mechanism of inhibition was that the FLAG antibody induced OCLN endocytosis, and thus sequestered it away from the plasma membrane where OCLN may be required to mediate HCV cell entry. Although visual analysis by fluorescence microscopy of the OCLN-GFP localization showed that incubation of FLAG antibody with OCLN EC2-F5 expressing 786-O cells did induce some intracellular relocalization of GFP signal (Figure 3A, fourth row), we do not believe this accounts for the major mechanism of entry inhibition. Firstly, FLAG antibody induced internalization of OCLN EC2-F5 was not absolute, as 45% of OCLN bound FLAG antibody was still detectable at the plasma membrane even after a 90 minute incubation (Figure 3B, ‘EC2-F5’). Furthermore, we observed that FLAG antibody induced an even greater degree of internalization of the OCLN EC2-F3 mutant (Figure 3B, ‘EC2-F3’), even though the antibody did not impair HCVpp infection of cells expressing this mutant (Figure 2B).

To further separate OCLN mutant internalization from the HCVpp blocking activity, we produced FLAG fragment antigen binding antibody (Fab), which is monovalent and thus not capable of cross-linking OCLN EC2-F5, which may be required to promote endocytosis. Indeed, although both full length FLAG IgG and Fab similarly inhibited HCVpp infection of OCLN EC2-F5 expressing 786-O cells (Figure 3C), the Fab fragment did not promote endocytosis of this protein (Figure 3A, bottom row). These results suggest that the primary mechanism of FLAG antibody inhibition is not due to OCLN internalization, but rather likely a consequence of the impairment of interactions between OCLN and viral or cellular partners essential for the HCV cell entry process.

### OCLN mutants complement OCLN silencing effects in HepG2 cells

**Figure 4:**
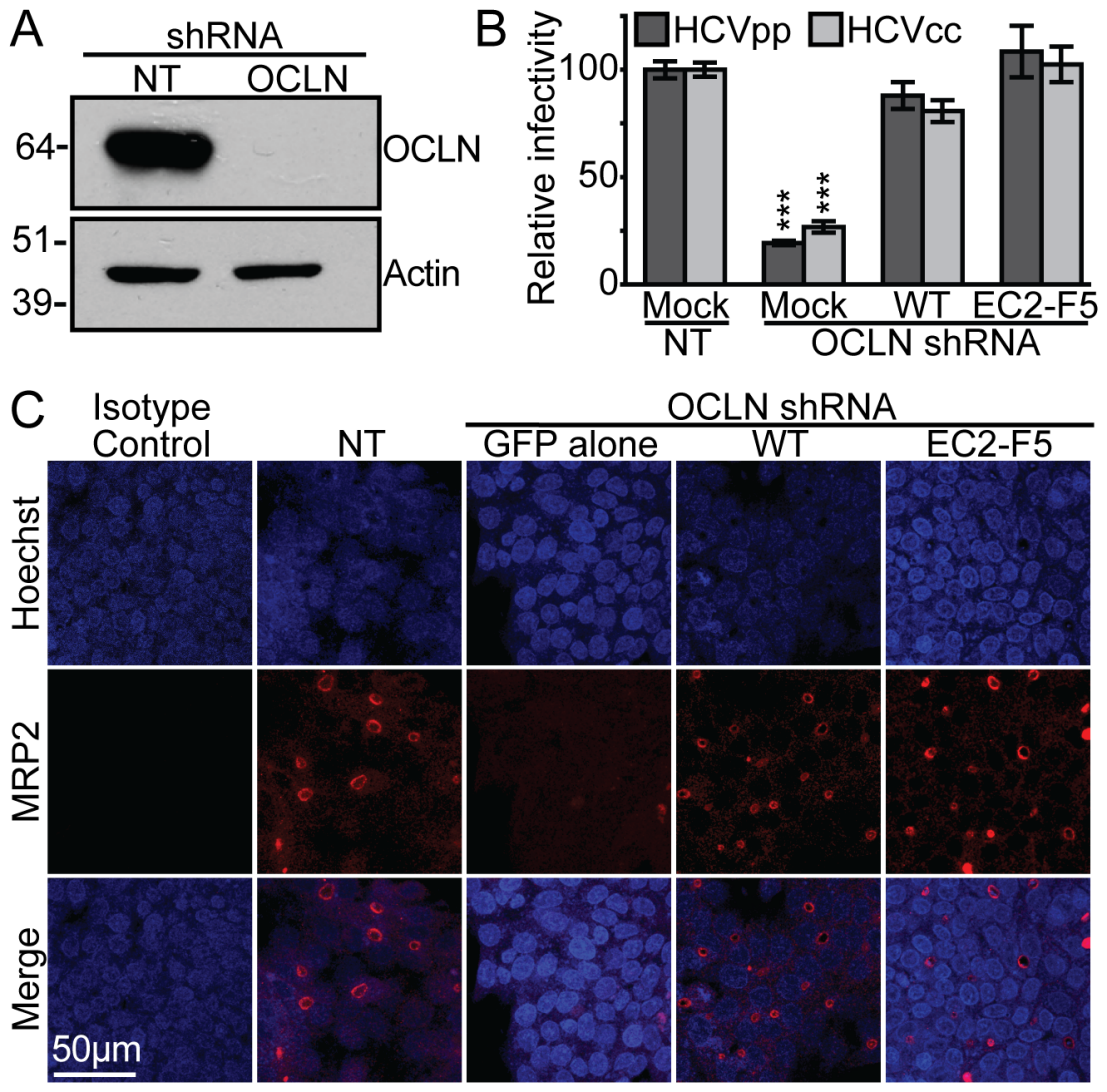
The OCLN EC2-F5 mutant can complement HCV entry and tight junction defects in OCLN silenced HepG2 cells. (A) Immunoblots for either OCLN or β-actin of lysates from HepG2+miR-122+CD81 cell populations transduced to express either no target (NT) or OCLN specific shRNAs. Approximate molecular weight (kDa) marker positions are indicated to the left of each blot. (B) These cells were either mock transduced (Mock), or transduced to express either shRNA-resistant wild type (WT) or the EC2-F5 OCLN mutant along with either NT or OCLN specific shRNAs, and then challenged with either HCVpp (dark) or HCVcc (light). ‘Relative infectivity’ refers to values that are normalized to parallel VSVGpp infections, to control for variations in cell number, and set relative to infections of cells expressing wild type human OCLN. (C) To analyze tight junction formation, cells transduced as in Figure 4B were grown for three days on collagen coated coverslips, and then stained for the bile canalicular marker MRP2 (red) or an isotype control and with Hoechst 33342 nuclear DNA stain (blue). A representative example of three independent experiments is shown. **P<0.01, *** P<0.001 (Mann-Whitney test).

We next investigated if the OCLN EC2-F5 mutant identified above could be used to conditionally regulate HCVcc infection of polarized HepG2 cells, which were engineered to support the entire HCV life cycle by expression of CD81 and the liver specific microRNA miR-122 [29]. Lentiviral expression of an shRNA targeting OCLN in HepG2+miR-122+CD81 cells efficiently silenced expression of endogenous OCLN (Figure 4A) and impaired infection with both HCVpp and HCVcc bearing the glycoproteins from the H77 genotype 1a and HC-J6 2a isolates, respectively (Figure 4B). Transduction with lentiviruses expressing shRNA resistant wild type and EC2-F5 mutant OCLN restored infection with both these viruses (Figure 4B). We found that OCLN silencing greatly reduced the ability of HepG2 cells to polarize, as gauged by staining of the apically localized ion channel multi-drug resistance protein 2 (MRP2) (Figure 4C, third column). Furthermore, expression of either wild type or EC2-F5 OCLN rescued this defect (Figure 4C, last two columns). These results indicate that OCLN is required for HepG2 cell polarization and confirms that the EC2-F5 mutant can support this phenotype.

**Figure 5:**
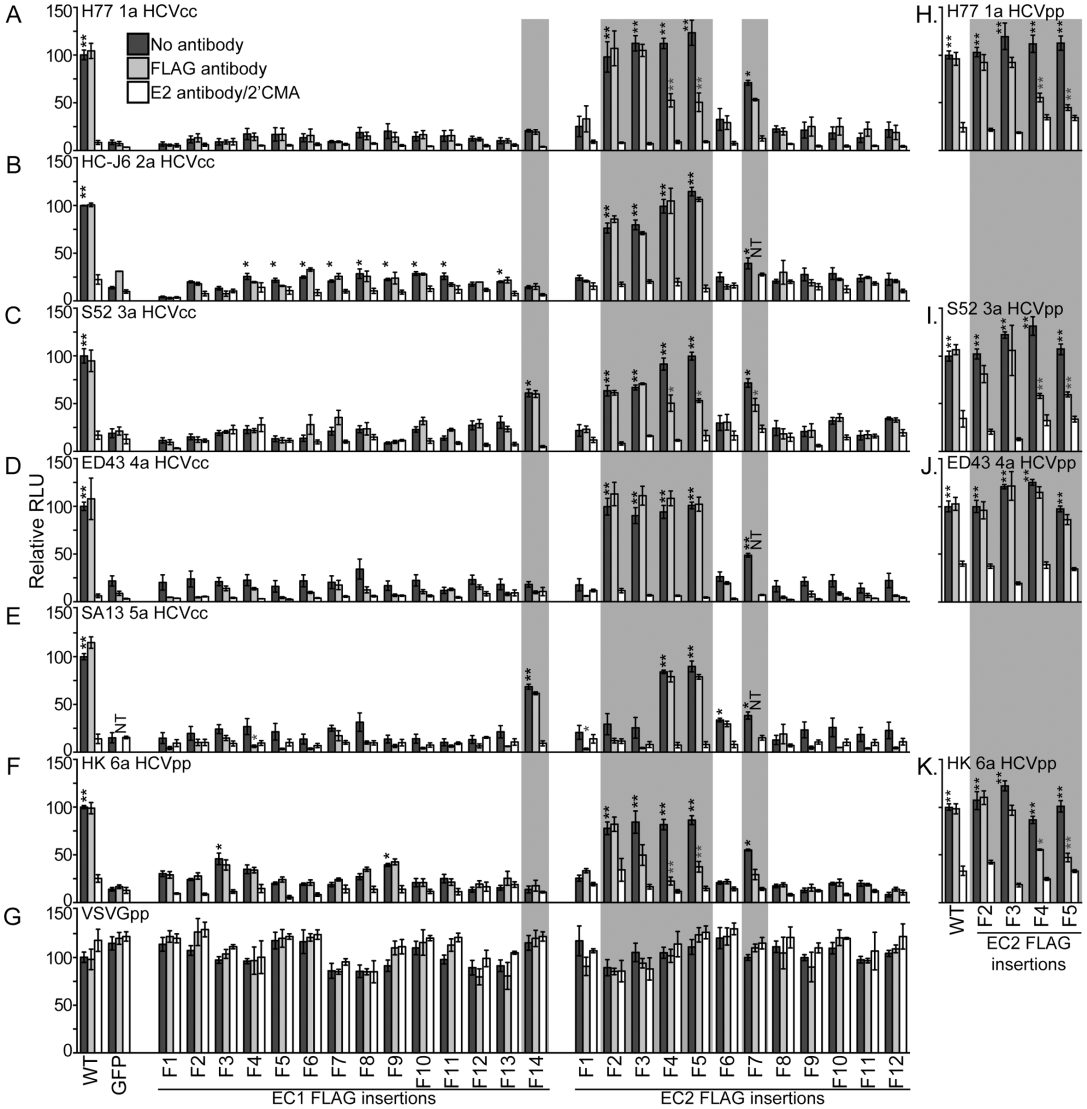
Mutant OCLN cell entry factor activities differ between HCV isolates. (A–G) HepG2+miR-122+CD81 cells that express an shRNA to silence endogenous OCLN expression or (H–K) 786-O cells were transduced to express either shRNA-resistant wild type OCLN (WT), GFP alone, or shRNA-resistant OCLN with the indicated FLAG insertional mutations and challenged with the indicated viruses in the absence of antibody (dark), in the presence of the M2 FLAG antibody (light), or, as positive inhibition controls, either an E2 monoclonal antibody, for HCVpp, or the HCV polymerase inhibitor 2′CMA, for HCVcc (white). Results are normalized to infection of cells expressing wild type OCLN for each virus in the absence of antibody. Gray boxes highlight mutants that exhibited different entry factor activities between isolates. *P<0.05, **P<0.01, *** *P*<0.001 (Mann-Whitney test). Black asterisks represent statistically significant differences between the indicated value and cells expressing GFP alone. Light gray asterisks represent statistically significant differences between infections of the same cells without and with antibody.

We next tested if HCVcc infection of HepG2+miR-122+CD81 OCLN EC2-F5 cells could be blocked by FLAG antibody. To our surprise, we found that infection of these cells with HCVcc bearing the structural proteins of the HC-J6 genotype 2a isolate was not impaired by FLAG antibody (Figure 5B). To determine if isolate-specific differences influenced OCLN entry factor usage and FLAG antibody blocking, HepG2+miR-122+CD81 cells with silenced endogenous OCLN were complemented with either wild type or the complete set of OCLN mutants tested above. These cells were challenged with HCVcc bearing the structural proteins from a range of HCV isolates, or HCVpp bearing the structural proteins of the genotype 6a HK isolate, for which we were unable to generate reasonable quantities of infectious HCVcc (Figure 5A–G). To control for residual luciferase enzyme in virus inoculums, parallel HCVcc infections were cultured in the presence of the HCV polymerase inhibitor 2′C-methyl-adenosine (2′CMA) [30] and HCVpp infections were incubated with the neutralizing E2 antibody AR3A [31].

While all viruses could utilize EC2-F4 and EC2-F5, FLAG antibody only blocked infection with HCVcc bearing the H77 genotype 1a and S52 genotype 3a HCV glycoproteins, and HCVpp bearing the genotype 6a HK glycoproteins (Figure 5). Other OCLN mutants displayed differences in HCV cell entry activities between HCV isolates. For instance, while most EC1 FLAG insertion mutants exhibited poor HCV cell entry activity, EC1-F14 was reasonably efficient at mediating infection of HCVcc bearing the S52 3a and SA13 5a HCV structural proteins (Figure 5C and E). While the EC2-F2 and EC2-F3 OCLN mutants functioned well for all viruses except SA13 5a HCVcc (Figure 5E), only HK 6a HCVpp infection of EC2-F3 expressing cells was inhibited, although not significantly, by FLAG antibody (Figure 5F). These results demonstrate that the ability of OCLN to participate in HCV entry into polarized cells can be conditionally regulated by addition of an antibody directed against an extracellular region of this protein. Furthermore, there are isolate specific differences in how HCV utilizes this protein to infect cells, which thus far has not been demonstrated for any of the other HCV cell entry factors. These isolate specific differences were not dependent on viral titer, as inoculums that had nearly equivalent titers (such as H77 1a and HC-J6 2a HCVcc) displayed different patterns of infectivity. In addition, such differences were not influenced by cell polarity and were also observed with HCVpp, as pseudoparticles bearing the glycoproteins from a subset of these isolates exhibited similar OCLN usage and antibody blocking patterns when tested in nonpolarized 786-O cell infections (Figure 5H–K).

### Analysis of the relative timing of entry factor usage

We used the above HepG2 cell system bearing the conditional OCLN EC2-F5 mutant to probe the point during cell entry that HCV utilizes OCLN. Although not the primary objective of this study, the following experiments are the first to examine the timing of action of any HCV cell entry factor in a polarized cell model. To do so, we conducted synchronized cell infection assays where the binding and post binding events were modulated by changing the temperature of the culture. First, virus was incubated with cells at 4°C, at which temperature virions only bind to the cell surface [19]. Unbound virus was then removed by washing with cold buffer, and we shifted the temperature by adding media prewarmed to 37°C to allow post-binding entry events, such as translocation across membranes, endocytosis, and fusion. Cells were then incubated at 37°C for two days, at which time the extent of infection was gauged by luciferase assay. We added blocking reagents targeting a range of HCV cell entry factors at various times before or after the binding phase. If a given entry factor had already been utilized by the virus, then the blocking reagent would be ineffective, thus indicting the point in the entry process that the entry factor is required.

**Figure 6:**
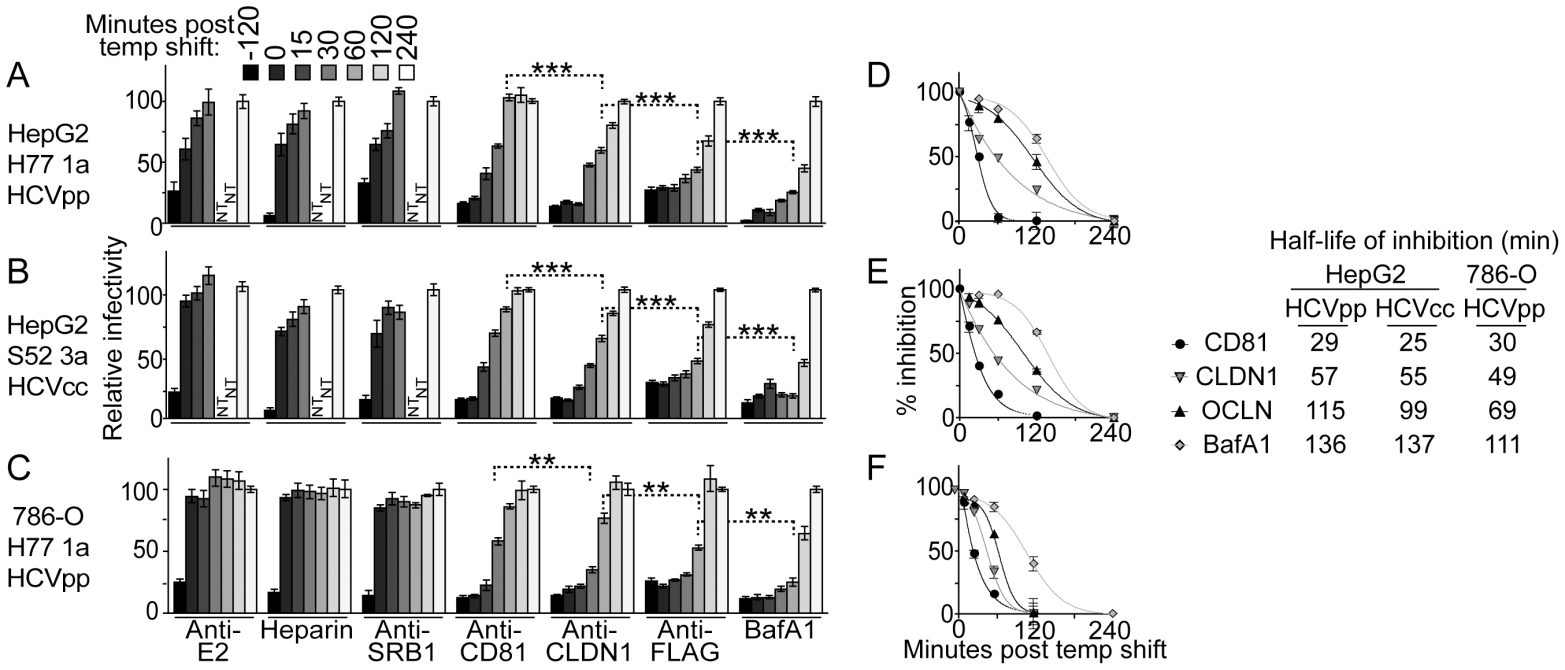
Analysis of the timing of entry factor usage during HCV cell infection. Synchronized infections of OCLN EC2-F5 mutant transduced (A+B) HepG2+miR-122+CD81 cells that express an shRNA to silence endogenous OCLN expression or (C) 786-O cells. Cells were challenged with either (A+C) H77 1a HCVpp or (B) S52 3a HCVcc. To probe the relative timing of each step in the entry process, the antibodies or drugs specified on the x-axes were added at the indicated times relative to the shift of the culture temperature to 37°C. For each inhibitor or antibody, results are relative to infection when inhibitor was added 240 min post temperature shift, at which point inhibition was no longer observed for any of the inhibitors. (D–F) To directly compare the kinetics of action of inhibitors that acted post-binding, the results from the graphs on the left were further normalized for each inhibitor to the amount of infectivity that occurred when that inhibitor was most and least active, which are set to 100% and 0% inhibition, respectively. Variable slope (CD81 and CLDN1) and Boltzmann (OCLN and BafA1) sigmoidal curves were fit to the % inhibition points and the equations for these curves were used to calculate the time of half maximal inhibition (t1/2) shown on the right. **P<0.01, *** *P*<0.001 (Mann-Whitney test).

Using this assay we found that both HCVpp and HCVcc exhibit similar entry factor usage patterns in HepG2 cells (Figure 6A and B). For both viruses, an E2 antibody [31], an SR-BI antibody [32], and heparin, which blocks virion association with GAGs, mainly inhibited host cell binding, as the majority of their blocking activity was lost at zero minutes post temperature shift. The remaining antibodies targeting CD81, CLDN1 [33], and OCLN all continued to strongly inhibit infection when added after the binding phase, indicating these factors are post-binding HCV cell entry factors. Furthermore, the kinetics of loss of inhibition with antibodies to CD81, CLDN1, and OCLN suggests that these entry factors are used in this order by the incoming particle. This finding is most easily illustrated by focusing on the 60 minute time point (Figure 6A and B, highlighted with dotted line brackets), when CD81 antibody inhibition is nearly completely lost, CLDN1 antibody inhibition is reduced by approximately half, and OCLN directed FLAG antibody still retains most of its blocking potential. Fusion within a low pH endosome, probed in parallel by adding bafilomycin A1 (BafA1), an inhibitor of vacuolar proton-ATPases that impairs intracellular vesicle acidification, took place even later in the entry process than usage of the tested cell entry factors. To assist in directly comparing the kinetics of action of each inhibitor that acted at a post-binding step, we plotted these data as percent inhibition relative to the observed maximum and minimum inhibitory activities of each agent (Figure 6D+E). The curves fit to these data points more clearly show the differences in the loss of inhibition, and were used to calculate the times of half maximal inhibition for each inhibitor, indicated to the right of the graphs. To determine if the timing of action of HCV cell entry factors was influenced by HepG2 cell polarity, we used this synchronized cell infection assay to study the kinetics of entry factor usage in nonpolarized 786-O cells expressing the OCLN EC2-F5 mutant. Although the overall rate of HCV cell entry appeared to be faster in these cells, we continued to observe sequential postbinding usage of CD81, CLDN1, and OCLN (Figure 6C+F). These results indicate that HCV cell entry likely occurs in distinct separable stages regardless of the degree of target cell polarity.

## Discussion

In this manuscript we describe our study exploring the role that OCLN plays in the HCV cell entry process. We screened a set of mutant OCLN proteins bearing epitope insertions in extracellular regions for those that exhibited normal HCV cell entry activity, yet that could be efficiently blocked by the addition of antibody. Our analysis of the entry factor activities of the tested OCLN insertional mutants, most of which were highly impaired, adds valuable insight into the possible mechanism by which HCV utilizes OCLN to enter host cells. All mutants except EC1-F2 and EC1-F12 were well expressed. Some mutants did not efficiently display the FLAG epitope on the cell surface, which suggests disrupted trafficking, although it is also possible that the epitope was simply buried within protein structure in these mutants. Our earlier analysis of OCLN HCV cell entry factor determinants showed that EC1 was highly tolerant of alanine substitutions [34], and other groups have found that the entire EC1 region can be removed without impairing HCV cell entry [35]. However here we found that all EC1 FLAG insertion mutants except EC1-F14 were highly impaired, which indicates that EC1 may indeed contain sequences that influence HCV cell entry. Our prior OCLN mutagenesis also led us to speculate that two absolutely conserved cysteine residues (C216 and C237) may disulfide bond to form a subdomain of EC2 whose sequence is essential for HCV cell entry [34]. In accordance with this hypothesis, most of the FLAG epitope insertions within this region (EC2-F6-F11) highly impaired HCV cell entry.

While six OCLN FLAG insertional mutants exhibited reasonable levels of HCV cell entry factor activity, only the EC2-F4 and EC2-F5 mutants functioned efficiently for all tested HCV isolates and could be blocked by FLAG antibody for at least a subset of these viruses. The lack of antibody inhibition with the EC2-F2 mutant may be a result of the epitope being buried within OCLN secondary structure and not because of poor expression of the mutant on the cell surface, as this mutant could be utilized by some HCV isolates. EC2-F3 on the other hand was well expressed and readily detectable on the cell surface by FLAG antibody, yet its HCV cell entry factor activity was not inhibited by antibody. The simplest explanation is that antibodies bound to this region do not obscure an interaction required for HCV cell entry. These results demonstrate that antibody must bind to particular regions of OCLN to inhibit HCV cell entry, which has implications for the development of antibody-based HCV antivirals targeting endogenous OCLN, and may explain why attempts to develop OCLN antibodies as therapeutics and research tools have been unsuccessful. While we show the EC2-F4 and F5 regions may be good antibody targets, other OCLN locations that did not tolerate insertions, such as the second half of EC2, may be as good if not better targets for antibodies that inhibit HCV infection and future efforts may wish to focus on these areas.

We hypothesize that FLAG antibody inhibits OCLN HCV cell entry activity by interfering with an interaction between OCLN and either a viral or cellular factor, rather than by inducing endocytosis. Although full length bivalent FLAG IgG induced some endocytosis of the OCLN EC2-F5 mutant, monovalent FLAG Fab also inhibited infection without inducing endocytosis. Furthermore, IgG induced a similar amount of endocytosis of the EC2-F3 mutant without impairing HCV cell entry of cells expressing this mutant, which suggests that the level remaining on the cell surface remained in excess of that which was required for entry activity. The fact that FLAG IgG did not block infection with some viruses encoding the structural proteins from particular HCV isolates further suggests that endocytosis is not involved in the mechanism of entry inhibition.

Our finding of differences between the efficiencies of both OCLN mutant utilization and FLAG antibody blocking for the tested HCV isolates is to our knowledge the first demonstration of isolate-specific HCV cell entry factor activity determinants. Furthermore, this finding supports a model where OCLN directly interacts with the incoming HCV particle. While the absolute requirement for OCLN, as well as previously studied sequence determinants, is shared between HCV isolates, we found that some epitope insertions were better tolerated by HCV bearing the structural proteins of particular isolates. These findings lead us to hypothesize that the HCV glycoproteins bind OCLN directly and isolates exhibit subtle differences in this interaction. If OCLN mediates HCV entry indirectly by interacting with other cellular proteins, we would not expect to see the isolate specific differences that we did unless these viruses exhibited substantially different cell entry pathways, which is unlikely. A direct interaction between viral glycoproteins and OCLN has not been demonstrated experimentally, perhaps due to technical issues with purifying correctly folded forms of OCLN and the HCV glycoprotein E1 or the E1/E2 heterodimer. The interaction may also be difficult to replicate experimentally because interactions between the HCV virion and other cell entry factors could first be required to prime the virion to be able to interact with OCLN.

To study the role of OCLN in HCV cell entry, we expressed the FLAG epitope insertion mutants in place of endogenous OCLN in HepG2 cells that were engineered to efficiently support the entire HCV life cycle by transduction to express CD81 and the liver specific microRNA miR-122 [29]. In these cells we found that silencing endogenous OCLN greatly inhibited polarization, indicating that this protein is required for tight junction assembly in HepG2 cells. Moreover, expression of wild type and EC2-F5 mutant OCLN proteins restored polarity. Although it does not rule out the possibility that EC2-F5 may still exhibit altered HCV cell entry factor activities or timing of action, it is reassuring that this mutant not only complements defects in HCV cell entry due to OCLN silencing, but can also restore the formation of tight junctions.

To understand the timeline of the HCV cell entry process, we used these EC2-F5 expressing HepG2 cells, in combination with a panel of inhibitors against a range of HCV cell entry factors, to analyze the relative timing of action of each factor. Our results support the model that HCV cell entry is a multistep process in which the incoming virus utilizes entry factors in succession that eventually results in fusion within an endosome. One potential caveat to keep in mind when interpreting these experiments is that antibody affinity may influence the precise timing of action determined for each entry factor. In general, since we used antibodies with reasonably high affinity and specificity it is unlikely that such differences would be severe or affect the apparent relative order of events. Antibodies targeting E2 and SR-BI, and soluble heparin only strongly inhibited entry when added during the binding phase of infection, indicating that these factors all play a role in HCV host cell binding. The role of SR-BI is somewhat controversial; the antibody we used has been previously shown to block at the cell binding stage [23], while another SR-BI antibody inhibits a postbinding event, more closely linked to the timing of action of CD81 [24]. This discrepancy likely reflects a dual role of SR-BI in HCV cell entry, where it may act to bridge binding and postbinding stages.

We found that antibodies targeting CD81, CLDN1, and the OCLN EC2-F5 mutant blocked infection when added either pre- or postbinding, indicating that these agents block postbinding HCV cell entry events. Furthermore, our analysis showed that these factors are used one after the other in the entry process. As with SR-BI, there is some controversy as to the precise timing of action of CLDN1. Our early analysis of CLDN1 kinetics with mutants bearing FLAG insertions in an extracellular region showed CLDN1 acts late in the entry process, after CD81 [14]. Subsequent studies with antibodies directed against wild type CLDN1 showed the timing of action of this factor essentially overlapped with CD81 [27]. It is possible that antibodies directed against various regions of CLDN1 demonstrate different kinetics of inhibition simply because they block functions required at different times, as speculated for SR-BI. Indeed, CLDN1 mutations have been shown to impair HCV cell entry at distinct stages, where some impair CD81 association while others block a later step [36], [37]. Here, we show that at least the CLDN1 antibodies we tested inhibit HCV cell entry with kinetics that is separable from those of CD81 antibodies. Of the entry factors examined, OCLN was required latest in the process. However, it remains to be examined where on the cell OCLN utilization actually takes place.

While this is the first study of the kinetics of HCV cell entry to use polarized cells, it is important to point out that although HepG2 cells are certainly more polarized than Huh-7 cells, these cells may not be as polarized as hepatocytes in vivo. For instance, in a given culture only up to 80% of HepG2 cells appear to form tight junctions [38]. We found that HCV cell entry required tight junction components later than other entry factors in both nonpolarized 786-O cells and at least partially polarized HepG2 cells. We consider it unlikely that the order of events would change in cells that exhibit higher degrees of polarity. Rather it is more probable that the relative timing of the process is simply more extended in cells where tight junction components are physically separated. Indeed, although it may be difficult to draw direct comparison between cell lines, we found that the kinetics of the postbinding events of the HCV cell entry process were faster in nonpolarized 786-O cells. To definitively determine how cell polarity influences the HCV cell entry process, future studies may require a range of HCV-permissive polarized cell systems and the development of techniques to modulate cell polarity without affecting expression levels or inducing internalization of tight junction components.

Our temporal map of the polarized cell HCV entry process agrees with the idea that it is a multistep process where the HCV virion uses readily available factors on the basolateral surface of hepatocytes prior to tight junction components that reside in physically difficult to reach cell surface locations. Such an entry pathway would be similar to that of coxsackievirus B, which first binds apically exposed decay-accelerating factor, followed by translocation of the virion into tight junction regions where it interacts with the coxsackievirus and adenovirus receptor prior to endocytosis [39]. In further support of such a model for HCV cell entry, CD81 antibodies or soluble E2 protein have been shown to initially bind CD81 diffusely across the surface of unpolarized Huh-7.5 cells, and then activate Rho GTPases to mediate actin-dependent relocalization of such CD81 complexes to regions of cell contact [40]. Full evaluation of this hypothesis will require a comprehensive visual examination of HCV entry into host cells by fluorescently labeling or immunodetecting virions and entry factors at various points during synchronized infections, similar to the recent analysis conducted by Coller et al. [21], except that we plan to utilize polarized cell systems and reagents such as those described here to target HCV cell entry factors.

## Materials and Methods

### Plasmid construction

As previously described [34], all OCLN proteins were expressed via lentiviral transduction from the context of pTRIP [41], [42], which is a self-inactivating lentiviral provirus that expresses no HIV proteins, but instead employs an internal CMV promoter to express cloned genes. The parental TRIP-hOCLN-PmeIGFP and negative control TRIP-GFP lentiviral plasmids have been previously described [34]. Two rounds of overlapping PCR were performed to generate OCLN products bearing single FLAG epitope insertions. As an example, to generate the OCLN EC2-F5 mutant, two PCR products were generated encompassing a region from the CMV promoter into the 5′ end of OCLN up to the EC2-F5 insert with the CMV-F oligo (5′ CGC AAA TGG GCG GTA GGC GTG) and an insert specific reverse oligo (5′ T GTC GTC GTC GTC CTT GTA GTC GAC TAT TTG TGA ACC ATA TAG AG , OCLN specific sequence is underlined) and a region from the EC2-F5 insert through the end of the OCLN coding sequence with a forward direction insert specific oligo (5′ C TAC AAG GAC GAC GAC GAC AAG CTT TAT GCC CTC TGC AAC CAA TT ) and an OCLN specific reverse oligo ME-O-438 (5′ ATA CCT GTC CAT CTT TCT TCG AGT TTT CAC AGC AAA G). These products were then reamplified with only the outside oligos, CMV-F and ME-O-438, to produce a single PCR product encoding a portion of the CMV promoter and OCLN coding sequence bearing the in frame FLAG epitope sequence, flanked by SalI and HindIII sites at the 5′ and 3′ ends, respectively. A similar strategy was used to generate each of the other FLAG epitope insertions (internal oligo sequences available upon request). These PCR products were subcloned into the TRIP-hOCLN-PmeIGFP plasmid by either BamHI or MscI and BstXI digestion, for either the EC1 or EC2 insertions, respectively. All PCR amplified sequences and cloning junctions were verified by sequence analysis.

### Cell culture and cell lines

293T, HepG2, and 786-O cells were grown in Dulbecco's Modified Eagle's Medium (DMEM; Gibco BRL Life Technologies, Gaithersburg, MD) with 100 U/ml penicillin, 100 µg/ml streptomycin (Mediatech, Inc, Manassas, VA), and 10% fetal bovine serum (FBS; Atlanta Biologicals, Lawrenceville, GA). HepG2+miR-122+CD81 cells were generated by transducing naïve cells with two lentiviruses: one encoding the puromycin resistance gene linked to the miR-122 genomic locus and another to express human CD81, as previously described [29]. These cells were grown in the same media described above supplemented with 1 µg/ml puromycin (Cellgro, Mediatech Inc) and grown on type I collagen (Gibco BRL Life Technologies) coated plates (30 µg/ml). To silence OCLN, these cells were transduced with a lentiviral pseudoparticle bearing the pLKO.1 lentiviral plasmid that expresses an shRNA specific for the 3′ UTR of OCLN mRNA (Broad Institute RNAi Consortium clone ID TRCN0000158804). As the OCLN coding sequence in the TRIP-hOCLN-PmeIGFP plasmids does not encode the OCLN 3′ UTR, these mRNAs are not susceptible to silencing with this shRNA.

### Virus generation and infection

Lentiviral pseudoparticle production, infections, and assays were performed as previously described [34]. Plasmids encoding the H77 1a (HCV isolate and genotype) [43], HCJ6 2a [44], S52 3a [44], ED43 4a [44], SA13 5a [44], and HK 6a [44] HCV envelope proteins to produce HCVpp have been previously described. HCVcc were produced as previously described [15]. Briefly, supernatants from Huh-7.5 cells transfected by electroporation with in vitro transcribed HCV genomic RNA were collected at 2, 3, and 4 days post transfection, filtered (0.45 µm pore size), and used for subsequent infections. All HCV plasmids encoded the nonstructural proteins from the genotype 2a JFH-1 isolate, whose strong replication capacity is linked to its ability to produce infectious particles [45]–[47] and were all bicistronic configurations with the HCV IRES directing translation of GLuc, and the EMCV IRES driving translation of the chimeric HCV genome. To produce HCVcc bearing the structural proteins of the genotype 1a H77 isolate, a chimeric HCV genome encoding the core through NS2 genes from the H77 isolate and three mutations that alleviate genetic incompatibilities between the H77 and JFH-1 proteins (K12N, I348S, and S1103T) [48] was used. The Jc1 plasmid, encoding the structural proteins from the HC-J6 isolate [49], was provided by Charles Rice (Rockefeller University). The S52/JFH, ED43/JFH, and SA13/JFH plasmids were kind gifts from Jens Bukh (Copenhagen University Hospital) [50]. The titers, determined by limited dilution assay based on NS5A staining of infected Huh-7.5 cells, as previously described [45], of each stock of virus bearing the indicated structural proteins used were: 4.0×10^4^ for H77 1a, 4.2×10^4^ for HC-J6 2a, 3.0×10^5^ S52 3a, 1.8×10^5^ for ED43 4a, and 2.8×10^4^ for SA13 5a tissue culture infectious dose per ml (TCID50/ml).

For both synchronized and nonsynchronized infections, 10^5^ cells were seeded on poly-L-lysine coated 24 well tissue culture plates 24 h prior to infection. For nonsynchronized infection experiments, cells were incubated with 250 µl of each virus dilution containing the indicated concentration of M2 antibody (Sigma, St. Louis, MO). HCVpp, HCVcc, and VSVGpp were diluted 1∶3, 1∶2, and 1∶5000 respectively, in media plus 4 µg/ml polybrene and 50 mM Hepes. Twenty-four hours postinfection, cells were washed 3 times with fresh media to remove Gluc protein present in the inoculum.

For synchronized infections, cultures were cooled to 4°C at t = −150 min prior to shifting the temperature to 37°C. At t = −120 min, media was replaced with 250 µl pseudoparticles or virus dilution containing 4 µg/ml polybrene and 50 mM Hepes, with or without inhibitor as indicated, and cultures were again placed at 4°C. At t = 0 min, cultures were washed twice with cold PBS, then fresh 37°C media, with or without inhibitor, was added and cultures were placed in 37°C tissue culture incubators. At indicated time points, inhibitors were added to the media at indicated concentrations. At 18 hours post infection, media was changed to fresh media without inhibitors. For both experiments, supernatants were harvested 2 days post infection in 25 µl cell culture lysis buffer and the expression of the luciferase reporter was measured as previously described [34].

### Antibodies and inhibitors

The M2 anti-FLAG antibody used for flow cytometry and inhibition assays was purchased from Sigma. The anti-E2 monoclonal antibody (clone AR3A) used to block HCVpp and HCVcc infections was provided by Mansun Law (Scripps Research Institute) [31]. The HCV polymerase inhibitor 2′C-methyl-adenosine (2′CMA) [30] was provided by Timothy Tellinghuisen (Scripps Research Institute). The anti-SRB1 antibody (mAb16-71) was provided by Alfredo Nicosia (CEINGE Biotecnologie Avanzate, Naples, Italy) [32]. The anti-CLDN1 monoclonal antibody (clone 5.16v5) was provided by Isidro Hötzel (Genentech, South San Francisco, CA) [33]. The anti-CD81 antibody (clone JS-81) was purchased from BD Pharmingen, (San Diego, CA); Bafilomycin A1 and heparin were purchased from Sigma.

### Immunoblot analysis

For immunoblot analysis of OCLN expression, cells were lysed in a volume of 1× SDS-PAGE sample buffer plus dithiothreitol (DTT) that was proportional to the approximate cell confluency. Cell lysates were passed through a 22-gauge needle several times and heated 5 minutes at 95°C. Equivalent volumes of lysate were immunoblotted with mouse monoclonal anti-OCLN (OC-3F10, Invitrogen, Carlsbad, CA) and mouse anti-β-actin antibodies (AC-15, Sigma, St. Louis, MO), to ensure analysis of comparable protein concentrations. For both antibodies, horseradish peroxidase (HRP) conjugated rabbit anti-mouse secondary antibody (Thermo Scientific, Rockford, IL) was used and detection was performed with Immobilon Chemiluminescent HRP (Millipore, Billerica, MA).

### Flow cytometry analysis

FLAG cell surface staining was performed on 786-O cells transduced to stably express various OCLN-GFP constructs. Cells were fixed in 4% PBS-paraformaldehyde (PFA) for 10 min. Staining was performed at 4°C for 20 min with the M2 mouse monoclonal anti-FLAG antibody (Sigma) and goat anti-mouse Alexa-647 antibody (Invitrogen) in PBS-1% BSA. For OCLN intracellular staining, cells were fixed in 4% PBS-PFA for 10 min. Staining was performed at room temperature for 45 min with the anti-OCLN rabbit polyclonal antibody (Invitrogen) and goat anti-rabbit Alexa-647 antibody (Invitrogen) in PBS-1% BSA – 0.05% saponin (Sigma). Isotype matched mAb or rabbit polyclonal antibody were used as negative controls. For quantification of GFP+ cells, cells were fixed in 4% PBS-PFA for 10 min.

For quantification of the FLAG induced endocytosis of the OCLN-GFP mutants, live cells were incubated for 15 min at 4°C with 10 µg/ml M2 anti-FLAG antibody (Sigma) in cold media. Antibody was washed out 3 times with cold media, then fresh 37°C media was added and cells were placed in 37°C tissue culture incubators. The cells were detached in PBS-EDTA 1 mM and fixed at the indicated time points with 4% PBS-PFA for 10 min. Cells were then cell surface stained with goat anti-mouse Alexa 647 antibody (Invitrogen). Fluorescence was analyzed by flow cytometry using a FACSCalibur (BD Biosciences, San Jose, CA) with FlowJo software.

### Microscopy

786-O cells transduced to stably express various OCLN-GFP constructs were seeded on poly-L-lysine coated 0.12 mm glass coverslips. Forty-eight hr post seeding, cell membranes and nuclei were stained with Alexa Fluor 594 conjugated Wheat Germ Agglutinin (WGA) and Hoechst (Invitrogen) respectively, according to manufacturer's directions, then fixed with 4% PFA and stained with an anti-GFP Alexa 488 (Invitrogen). HepG2 cells were seeded on collagen coated 0.12 mm glass coverslips. Ninety-six hours post seeding, cells were fixed with 4% PBS-PFA and stained for MRP2 using an anti-MRP2 monoclonal antibody (clone M2 III-6, Abcam, Cambridge, MA) and anti-mouse Alexa 568 secondary antibody (Invitrogen) in PBS containing 0.2% BSA and 0.05% saponin and for the nucleus, with Hoechst (Invitrogen) in HBSS media. All slides were imaged on a Leica TCS-SP5 DM laser scanning confocal microscope. Images were processed and analyzed using the ImageJ program (http://rsb.info.nih.gov/ij/). Representative images show Z-sections of approximately 0.4 µm.

### Statistical analysis

Data were analyzed for statistical significance using Prism software (GraphPad Software) using the Mann-Whitney test. A p-value≤0.05 was considered significant. Values in graphs represent the mean and standard error of greater than three independent experiments, each performed in triplicate.
